# Oxidized Polyethylene Wax as a Potential Carbon Source for PHA Production

**DOI:** 10.3390/ma9050367

**Published:** 2016-05-13

**Authors:** Iza Radecka, Victor Irorere, Guozhan Jiang, David Hill, Craig Williams, Grazyna Adamus, Michal Kwiecień, Adam A. Marek, Jan Zawadiak, Brian Johnston, Marek Kowalczuk

**Affiliations:** 1 School of Biology, Chemistry and Forensic Science, Faculty of Science and Engineering, University of Wolverhampton, Wolverhampton WV1 1LY, UK; I.Radecka@wlv.ac.uk (I.R.); V.Irorere@wlv.ac.uk (V.I.); Guozhan.jiang@wlv.ac.uk (G.J.); D.Hill@wlv.ac.uk (D.H.); C.Williams@wlv.ac.uk (C.W.); B.Johnston@wlv.ac.uk (B.J.); 2 Centre of Polymer Chemistry, Polish Academy of Sciences, Zabrze 41-800, Poland; Grazyna.Adamus@cmpw-pan.edu.pl (G.A.); mkwiecien@cmpw-pan.edu.pl (M.K.); 3 Department of Chemical Organic Technology and Petrochemistry, Silesian University of Technology, Gliwice 44-100, Poland; adam.a.marek@polsl.pl (A.A.M.); jan.zawadiak@polsl.pl (J.Z.)

**Keywords:** polyhydroxyalkanoates, oxidized PE wax, medium chain length PHA (mcl-PHA), *Ralstonia eutropha* H16, mass spectrometry

## Abstract

We report on the ability of bacteria to produce biodegradable polyhydroxyalkanoates (PHA) using oxidized polyethylene wax (O-PEW) as a novel carbon source. The O-PEW was obtained in a process that used air or oxygen as an oxidizing agent. *R. eutropha* H16 was grown for 48 h in either tryptone soya broth (TSB) or basal salts medium (BSM) supplemented with O-PEW and monitored by viable counting. Study revealed that biomass and PHA production was higher in TSB supplemented with O-PEW compared with TSB only. The biopolymers obtained were preliminary characterized by nuclear magnetic resonance (NMR), gel permeation chromatography (GPC), differential scanning calorimetry (DSC), and thermogravimetric analysis (TGA). The detailed structural evaluation at the molecular level was performed by electrospray ionization tandem mass spectrometry (ESI-MS/MS). The study revealed that, when TSB was supplemented with O-PEW, bacteria produced PHA which contained 3-hydroxybutyrate and up to 3 mol % of 3-hydroxyvalerate and 3-hydroxyhexanoate co-monomeric units. The ESI-MS/MS enabled the PHA characterization when the content of 3-hydroxybutyrate was high and the appearance of other PHA repeating units was very low.

## 1. Introduction

Petrochemical-based plastics have become an integral part of our daily lives since their first industrial production in the 1940s. They are durable, lightweight, inexpensive, strong, and easy to handle, possessing properties that make them very useful in agricultural, industrial, and domestic applications [1,2]. However, these properties also make them susceptible to excessive use and irresponsible disposal [3]. Thus, plastic waste makes up a huge part of municipal solid waste (MSW), globally. In Europe, it is estimated that about 7% by mass of generated MSW are plastics. This percentage is even higher in the USA and China both having 11.8% and 14% by mass, respectively [4,5].

Furthermore, it is estimated that plastics contribute up to 80% of marine debris causing serious environmental and health problems worldwide [5]. The environmental and health impact of these plastic wastes are severe and have been extensively discussed by previous authors [6]. Polyethylene (PE) is known to be the most manufactured petrochemical polymer making up over 29% of global petrochemical plastic production [7] and with only 10% of plastic waste presently being recycled [8], there is a huge demand for alternative uses of these waste materials to reduce the environmental burden they have on today’s society.

PE is a chemosynthetic polymer derived mostly from fossil fuels and recently also from renewable sources [9]. The very rich carbon backbone of PE makes it a potential cradle of carbon for microbial utilization in the production of the biodegradable polyhydroxyalkanoates (PHA). PHA are readily biodegradable, biocompatible, and non-toxic biopolymers that are synthesized intracellularly by different bacterial species from soluble carbon rich substrates in the presence of an excess amount of carbon and a limiting nutrient [10,11,12]. In addition, they also possess properties similar to petrochemical plastics, thus making them potential substitutes for non-biodegradable polymeric materials [10]. The applications of PHAs in various sectors of our present day society are diverse and have been reviewed by various authors [13,14,15,16]. Generally, the cost of production of biodegradable plastics are higher than the conventional plastics since most of the processes are still in the developmental stage. Thus, the decrease of waste treatment cost of conventional plastics through PHA recovery seems to be one possible solution [17].

The use of waste polyethylene as carbon source for PHA production brings in a double advantage of reducing the environmental impact of polyethylene plastic and producing an environmentally friendly and biodegradable substitute. However, the highly stable C-C and C-H covalent bonds of PE coupled with its very high molecular weight and hydrophobicity makes it difficult for microbial attack and utilization [11,18]. Recycling technologies of PE are restricted either to mechanical recycling or energy recovery via incineration. Oxidation of PE can generate a valuable feedstock in the form of oxidized PE wax rich in hydrocarbons. Some microbes should be able to use this highly complex substrate for synthesis of the added value biodegradable polymers such PHA, therefore, the conversion of PE to a high value product should lead to higher level of PE recycling [7]. This process should also have an impact on the production cost of PHA as the use of waste O-PEW feedstock for microbes accumulating PHAs can lead to their greater economic viability and sustainability.

PE waxes are very important materials in many types of industry. One of the most important groups of synthetic wax are polar polyethylene waxes, which are obtained in the oxidative degradation process. The process occurs with chains scissoring and the formation of oxygen-containing groups (hydroxyl, carbonyl, carboxyl, ester), according to the mechanism of free radical chain [19]. Polar polyethylene waxes are primarily used as a component of aqueous emulsions with many applications including additives for paints and varnishes [20], lubricants e.g., in the polyvinyl chloride (PVC) manufacture [21], and many others. Their application properties depend on the type of raw materials used (low-density polyethylene (LDPE), high-density polyethylene (HDPE)) and, thus, the method of production, which may be carried out as a process in melt [21,22], aqueous dispersion [23,24], or solid phase [25,26].

The model studies on oxidized polyethylene powder as a filler for polycaprolactone blends revealed that pre-oxidized PE powder in combination with surfactant and pro-oxidant greatly accelerated the degradation rate of poly(*ε*-caprolactone), PCL, as revealed by molecular weight decrease during low temperature thermo-oxidative aging [27]. Recently, the polar O-PEW was used as an addition to the polyhydroxybutyrate (PHB) biopolyester improving the mechanical properties of the prepared polymeric materials as confirmed by thermo-mechanical studies [28]. The beneficial composition was found for the blend containing up to 10% (*w*/*w*) of oxygenated PE wax, and biodegradation studies conducted under both laboratory and an industrial composting conditions revealed that the O-PEW accelerated the biodegradation rate of the PHB blend.

Thus, our hypothesis is that O-PEW could be a potential source of carbon for microbial PHA production and also act as the PHA blend component which could accelerate their biodegradation.

Simple pyrolysis of PE in the absence of air generates a complex mixture of low molecular weight paraffin with carbon chain lengths from C8 to C32 (PE pyrolysis wax). Guzik *et al.* used the PE pyrolyzed wax for long side chain PHA production and *Pseudomonas aeruginosa* PAO-1 was found to accumulate the highest level of PHA to almost 25% of the cell dry weight when supplied with this type of PE pyrolysis wax in the presence of rhamnolipids [7].

Here, we propose the use of O-PEW for PHA production. PE oxidation introduces carbonyl and hydroxyl groups into the polymer backbone that are more easily cleaved by microbes [29]. This also improves PE’s hydrophilic properties and reduces its chain length, thus further improving their potential for microbial utilization as a carbon source for PHA production. Furthermore, the process of oxidation improves the dispersive properties of PE wax in the fermentation media and makes them more accessible to microbes. Bio-utilization of O-PEW follows a process that results in the production of fatty acids which can then be *β*-oxidized within the cell [18].

*Ralstonia eutropha* (previously known as *Cuprivadus necator* or *Wausternia eutropha*) is a gram negative bacterium that is known to accumulate PHB as insoluble granules within their cells when nutrients other than carbon are limiting. However, it has been also reported that in some cases PHA production by this organism can also occur under nutrient-rich conditions [12,30]. *Ralstonia eutropha* has been reported to utilize a wide range of carbon source including fatty acids for PHA production [10,31]. This bacterium can accumulate 80% to 85% PHA per dry cell weight with about 8–12 PHB granules per cell. PHA granules are then extracted from this dried biomass via solvent extraction mainly with chloroform and precipitated in ethanol [32] or through other extraction techniques. Thus, in this research we report the production of PHA by *Ralstonia eutropha* H16 incubated in two different microbiological media (nitrogen rich tryptone soya broth and nitrogen-limited basal salts medium [12]) supplemented with O-PEW as the carbon source. This to the best of our knowledge is the first report showing the use of oxidized polyethylene wax as a carbon source for PHA production.

## 2. Results

### 2.1. O-PEW Preparation and Characterisation

**Figure 1:**
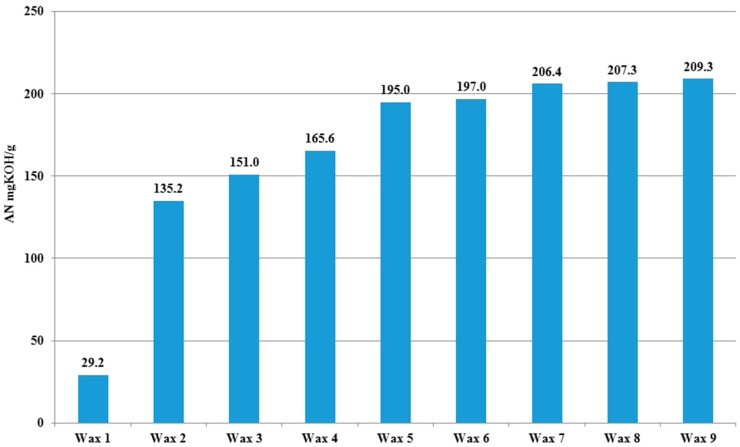
Acid number (AN) of O-PEW obtained in the melt phase oxidation process.

The oxidation process of PE causes partial polymer degradation and changes to the surface properties of the examined material [33]. For the purpose of this study we performed the oxidation process in melt phase, which is the best option for selected low molar mass PE standard containing no additives. In order to avoid the introduction of impurities which may have had a negative influence on the bacteria, no initiators or catalysts were used. Instead of that, the ozone was applied to increase the rate of the process which can be performed with repeatability [24,25]. Using this methodology of oxidation the acid value of O-PEW can be controlled by the process time as well as content of ozone in the oxidizing mixture (Figure 1).

Products with lower acid numbers were obtained in shorter processes carried out in an oxygen atmosphere with lower ozone content. The first wax was obtained without ozone addition after 18 h. Waxes 2–4 were oxidized with different ozone concentrations for 18 h. The waxes 5–6 and waxes 7–9 differed in processing time. Together with the increase in the acid number, the oxidized waxes become more yellowish and soft, because of the higher degree of degradation. For the shake-flasks experiments, the O-PEW with acid number (AN) = 197 was used as an optimum. All O-PEWs with an AN around 200 were easy to mix into tryptone soya broth (TSB) or basal salts medium (BSM), on the contrary waxes with an AN lower than 165 were very difficult to mix with the microbial growth media.

### 2.2. Bacterial Growth and PHA Separation

**Figure 2:**
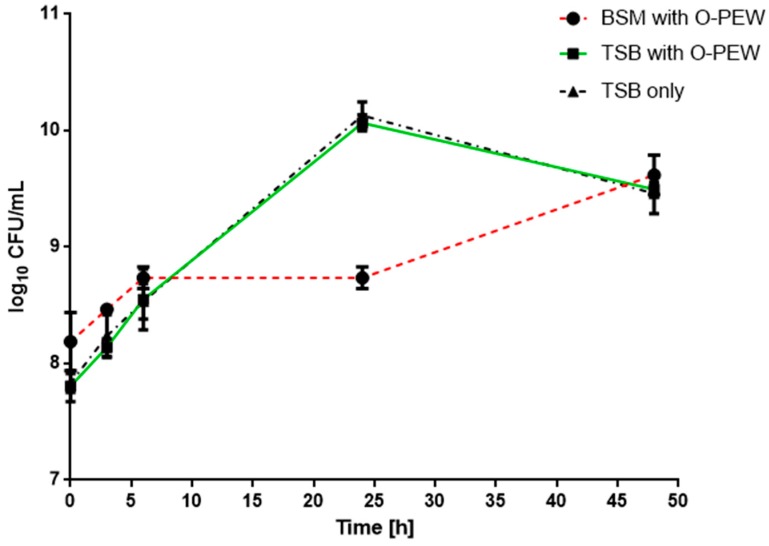
Small-scale shake-flasks experiments of *R. eutropha* H16 with 5 g/L of O-PEW in TSB (green line, black squares) or BSM (red line, black circles) and TSB only (black line, black triangles). Viable count (log_10_ CFU/mL) data points at time 0, 3, 6, 24, and 48 h are the arithmetic means of triplicates, while error bars represent the standard error (SE) of the mean.

The growth of *R. eutropha* H16 in TSB (control) and TSB or BSM supplemented with O-PEW is presented in Figure 2.

The bacteria inoculated at around 7.8 log_10_ CFU/mL grew to a maximum cell number of 10.13 log_10_ CFU/mL and 10.07 log_10_ CFU/mL in the TSB only and the wax supplemented TSB experiments respectively after 24 h of incubation. After this 100-fold increase at 24 h of incubation, no further cell growth was observed and the number of cells in both cultures decreased by 0.5 log_10_ CFU/mL by 48 h. In BSM with O-PEW the bacteria inoculated at around 8.18 log_10_ CFU/mL grew much slower reaching a cell number of 8.7 log_10_ CFU/mL after 24 h. A maximum cell number of 9.61 log_10_ CFU/mL was observed after 48 h. All results were statistically analyzed using two way ANOVA test. A significant difference was found between bacterial growth in BSM with wax and both TSB with and without wax after 24 h of incubation (*p* < 0.05), while no significant difference was found after 48 h. No bacterial growth was observed in BSM only (data not shown).

The average cell dry weight (CDW) and the average PHA yield after 48 h of growth in the microbiological growth media are presented in Table 1. It was found that PHA was only produced in TSB and that the presence of O-PEW both increased biomass and PHA production.

### 2.3. PHA Identification and Characterisation

The results of the molar mass measurements from the obtained polyesters, estimated by GPC experiments with polystyrene standards, revealed that the number-average molar mass (*Mn*) in the cases of PHA polyesters obtained using TSB as the carbon source was found to be in the range of 52,000 g/mol. The *Mn* for polyesters using TSB/O-PEW as a carbon source was higher within the range of 220,000 g/mol.

**Figure 3:**
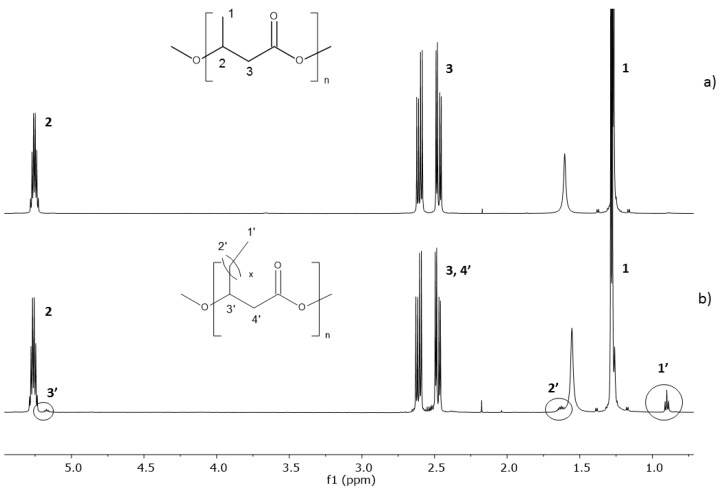
^1^H-NMR spectra of the polyesters obtained via fermentation of *R. eutropha* H16 with the use of (**a**) TSB; and (**b**) TSB/O-PEW as an additional carbon sources.

The ^1^H-NMR spectra of the polyester obtained using TSB only and TSB/O-PEW are presented in Figure 3a,b, respectively. In both spectra, the signals corresponded to the protons of the 3-hydroxybutyrate repeating units (labelled 1, 2, and 3) were observed. Moreover, the additional signals (at *δ* = 0.9, 1.63, 5.17), were presented in Figure 3b.

Such signals observed in the spectrum in Figure 3b are characteristic for polyhydroxyalkanoates containing in their structure monomer units with longer aliphatic groups in *β*-position [34]. Based on integration of the signals acquired, the content of other monomer units other than 3-hydroxybutyrate (3-HB) was estimated in the range of 3 mol %. However, unequivocal determination of the structures of the monomer units other than 3-hydroxybutyrate was not possible using ^1^H-NMR analysis due to the overlapping of the signals corresponding to the protons of aliphatic groups in the *β*-position. The ^13^C-NMR spectra for both polyesters obtained were recorded. However, undoubtedly due to the low content of the monomeric units with longer aliphatic chains in the *β*-position, only the signals of protons corresponding to the 3-hydroxybutyrate units were observed for the sample obtained in the presence of TSB with O-PEW as the carbon source.

**Figure 4:**
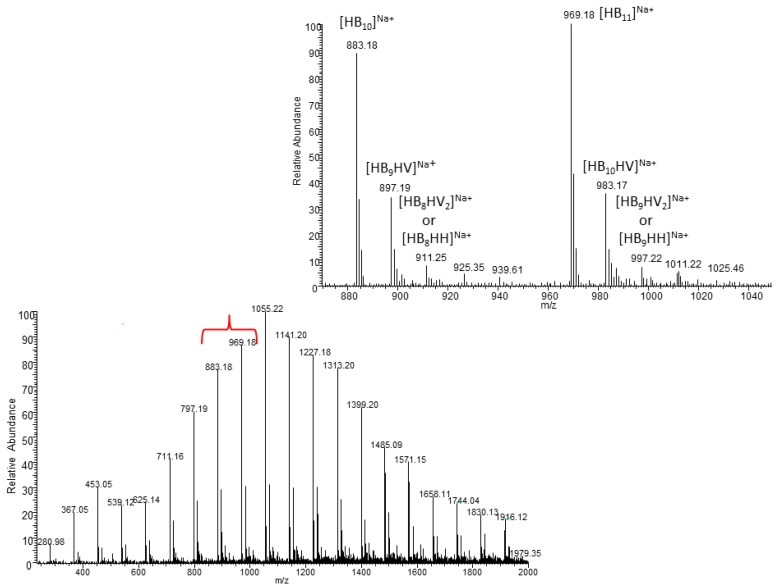
The ESI-MS spectrum (positive-ion mode) of the PHA oligomers, obtained via partial thermal degradation of the biopolyester obtained using TSB/O-PEW as a carbon source, together with an extended spectral range of *m*/*z* 870–1050.

The ESIMS spectrum of the oligomer resulting from Elimination Unimolecular conjugate Base (E1cB) degradation of the biopolyester obtained with the use of TSB/O-PEW is presented in Figure 4.

**Scheme 1:**
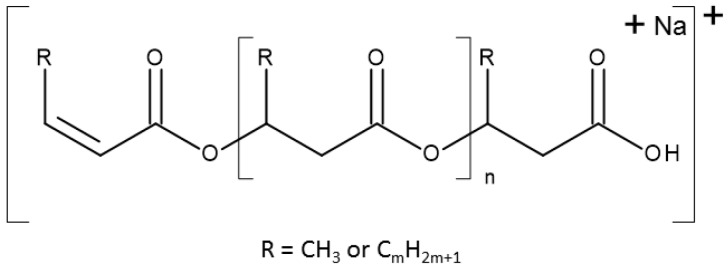
General formula of ions observed in ESI-MS spectra.

The general structure of the ions presented in the ESI-MS spectrum is shown in Scheme 1.

**Figure 5:**
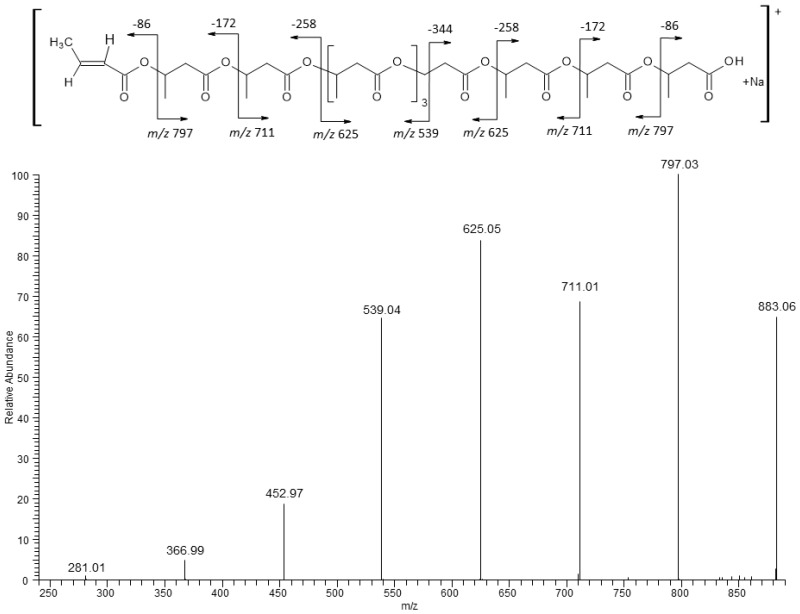
ESI-MS/MS spectrum obtained for the selected sodium adduct ion of oligomers in [HB_10_ + Na]^+^ *m*/*z* 883.

In order to verify the structure of the individual ions belonging to the clusters, the ESI-MS/MS fragmentation experiments were performed for selected cluster ions. The ESI-MS/MS product ions spectrum for the selected sodium adducts of oligomers [HB_10_ + Na]^+^ at *m*/*z* 883 is presented in Figure 5.

**Figure 6:**
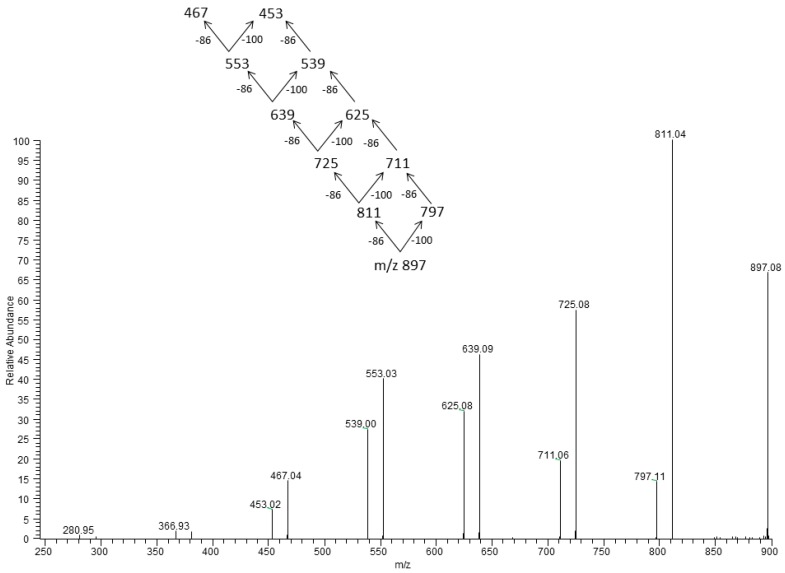
ESI-MS/MS spectrum obtained for the selected sodium adduct of oligomers [HB_9_ HV + Na]^+^ at *m*/*z* 897.

In Figure 6 the ESI-MS/MS spectrum for the selected ion [HB_9_ HV + Na]^+^ at *m*/*z* 897, which contains nine 3-HB and one 3-HV units, is presented.

**Figure 7:**
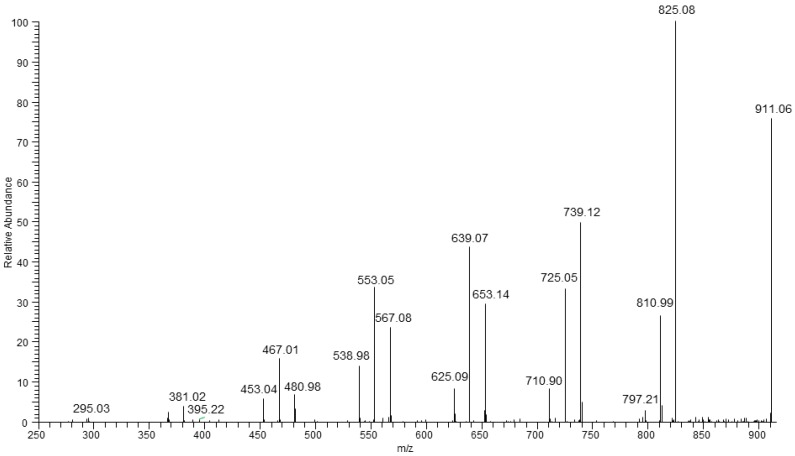
ESI-MS/MS spectrum obtained for the selected sodium adduct of oligomers at *m*/*z* 911.

Figure 7 shows the three fragmentation paths that were observed for the parent ion at *m*/*z* 911.

The thermal properties of the obtained PHA were determined by differential scanning calorimetry (DSC) and thermogravimetric analysis (TGA). The results are summarized in Table 2.

DSC analysis included values of glass transition temperature (*T_g_*), melting temperature (*T_m_*), and crystallinity degree (*C*) calculated according to the experimental section. The *Tg* for samples from TSB and TSB/O-PEW were equal to 6.2 °C and 1.9 °C, respectively. The melting points (*T_m_*) for the obtained polyesters were equal to 171 °C for PHB from TSB and 166 °C for polyester from TSB/O-PEW. The crystallinity degrees were equal to 47% for PHB from TSB only and 42% for PHA from TSB/O-PEW. The TGA results are presented as the maximum decomposition temperature (*T*_max_).

## 3. Discussion

A major problem of waste and environmental pollution is that plastics produced by the petrochemical industry are not biodegradable and therefore accumulate in the environment. The conversion of polyethylene (PE) to oxidized PE wax (O-PEW) and its use as carbon source for bacterial PHA production can be an attractive alternative for producing environmentally friendly and biodegradable polymers. The oxidation process can have an impact on the properties (AN) of the PE wax (Figure 1). Previous experiments have shown that polyethylene (PE) can have negligible antimicrobial properties against bacteria. Thus, Zhang *et al.* noted that PE has very low antimicrobial properties [35] and, Seyfriedsberger and his colleagues showed that linear low density polyethylene (LLDPE) also had very low antimicrobial activity against Gram-positive *Staphylococcus aureus* and no antimicrobial activity against gram negative *Escherichia coli* [36]. Gregorova *et al.* reported that PE had no antimicrobial property against either *S. aureus* or *E. coli* [37]. In this study cell growth analysis showed that O-PEW 17 with AN = 197 does not affect the growth of *R. eutropha* H16 in both media TSB or BSM (Figure 2). Our results suggest that O-PEW is metabolized presumably via *β*-oxidation leading to enhanced cell growth of *R. eutropha* H16.

PHA yield and percentage PHA per cell dry weight (1.24 g/L and 33.8%, respectively) were higher in TSB supplemented with O-PEW compared to pure TSB (0.39 g/L and 17%, respectively). Growth and metabolism of O-PEW was confirmed by *R. eutropha* H16 being able to grow in BSM with oxidized polyethylene wax as the only carbon and energy source. Growth of *R. eutropha* H16 in BSM with O-PEW did not produce PHA with the 48 h culture period (Table 1). These results suggest the addition of O-PEW into TSB has an effect upon the production of PHA. Stress and the availability of carbon sources are known factors that stimulate PHA accumulation within bacterial cells [10]. The presence of accessible carbon sources, such as fatty and carboxylic acids from O-PEW for microbial use means that more carbon is present in the wax supplemented cultures in a form that promotes synthesis of PHAs. It may be expected, that in the presence of a biosurfactant [7] these wax carbon sources would be more accessible, leading to more PHA accumulation. The amount of nitrogen available in the media can influence bacterial growth and accumulation of PHA. In this study higher concentration of biomass and higher concentrations of PHA were obtained in TSB (high nitrogen content) than in BSM (low in nitrogen) which agrees with observations reported by Verlinden *et al.* [12] when waste frying oil was used to produce polyhydroxybutyrate (PHB). Addition of O-PEW stimulated further bacterial growth that may have led to increased nutrient utilization and eventually nutrient limitation, resulting in better production of PHAs.

Today mass spectrometry complements in many ways the structural data provided by NMR [34]. Development of soft ionization techniques in mass spectrometry made the attempt to solve the difficult question regarding the molecular structure of copolymers more likely. Thus, structural studies of biodegradable copolymers with the use of multi-stage electrospray mass spectrometry (ESI-MS^n^) were performed [38]. This mass spectrometry technique was applied to determine the co-monomer unit composition and composition distribution in bacterial PHA copolymers based on the analysis of their oligomers obtained by partial depolymerization (Figure 4). For the purpose of this study, the controlled thermal degradation of polyesters obtained, induced by sodium acetate, was performed according to the procedure described in the reference [39]. This type of E1cB degradation leads to PHA oligomers with unsaturated and carboxylic end groups.

The ESI-MS spectrum of oligomer, obtained *via* partial thermal degradation of the biopolyester obtained using TSB (without wax) as a bacterial growth medium, indicated that the presence of singly charged ions contained 3-hydroxybutyrate (3-HB) repeat units only. However, the ESI-MS spectrum of oligomer derived from PHA obtained using TSB/O-PEW (presented in Figure 4) consists of a series of clusters with singly charged ions. The ions were separated due to their different degrees of oligomerization and composition. The differences between the *m*/*z* value of the most intensive signals are equal to 86, which corresponds with the molar mass of the 3-hydroxybutyrate (3-HB) repeat units. However, in the extended spectral range at *m*/*z* 870–1050 the mass difference between the *m*/*z* values of ions within each cluster (e.g., *m*/*z* 897, 911, 925, and 939) is equal to 14 Da, which corresponds to the difference between the molar mass of the individual co-monomeric units with longer side chains.

Based on the mass assignment of singly charged ions observed in the mass spectrum the structure of the end groups and repeating units can be inferred. The clusters of singly charged ions observed in the mass spectrum in Figure 4 corresponds to the sodium adducts of individual co-oligoester chains composed of 3-hydroxybutyrate (HB), 3-hydroxyvalerate (HV) and/or 3-hydroxyhexanoate (HH) co-monomeric units and were terminated by unsaturated and carboxylic end groups.

The ESI-MS/MS product ions spectrum for the selected sodium adducts of oligomers [HB_10_ + Na]^+^ at *m*/*z* 883 were shown in Figure 5. Fragmentation of this ion, which resulted from the random breakage of ester bonds along the both sides of the oligomer chain, led to the formation of only one set of oligo(3-hydroxybutyrate) product ions at *m*/*z* 797, 711, 625, 539, 452, 366, and 281 terminated by carboxyl and crotonate end groups. The first ion from this series at *m*/*z* 797 was created by the displacement of neutral molecule of crotonic acid (86 Da) from both ends of the parent ion at *m*/*z* 883. The fragmentation spectrum of this ion, confirms that the most intensive ions in the clusters correspond to sodium adducts of 3-hydroxybutyrate oligomers. In Figure 6 the ESI-MS/MS spectrum for the selected ion [HB_9_ HV + Na]^+^ at *m*/*z* 897 (which contains nine 3-HB and one 3-HV units) was presented. In that case, the breakage of ester bonds along the oligomer chains led to the formation of two series of product ions at *m*/*z* 811, 725, 639, 553, 467, 381 and at *m*/*z* 797, 711, 625, 539, 453, 366. Thus, the product ion at *m*/*z* 811 corresponded to the oligomer formed by the loss of crotonic acid (86 Da) while the product ion at *m*/*z* 797, could have been formed due to the loss of valeric acid (100 Da), as seen from the fragmentation pathway in Figure 6. Thus, the fragmentation spectrum acquired for the precursor ion at *m*/*z* 897 confirms the presence of 3-hydroxybutyrate and the 3-hydroxyvalerate co-monomer units in polyester chains. Moreover, such a fragmentation pathway indicates that the 3HV unit is randomly distributed along the oligomer chain [38,40,41].

In Figure 7, three fragmentation paths were observed in the case of ESI-MS/MS spectrum of the parent ion at *m*/*z* 911 (which may have corresponded to the isobaric ions contained in two HV units or one HH unit [HB_8_ HV_2_ + Na]^+^ or HB_9_ HH + Na]^+^, respectively. These two series at *m*/*z* 825, 739, 653, 567, 480, 395, and at *m*/*z* 811, 725, 639, 553, 467, 381 are formed in the same way as described for fragmentation of ion at *m*/*z* 897 (Figure 6). The third series at *m*/*z* 797, 711, 625, 539, 453, 367 was also observed. The first ion in this series is formed by expulsion of 2-hexenoic acid (114 Da) indicating, unequivocally, the presence of HH unit in the oligomer chain. Thus, the fragmentation experiment carried out for the ion at *m*/*z* 911 indicates the presence of more than two co-monomeric units in this oligomer. The ESI-MS/MS spectrum of sodium adduct at *m*/*z* 911 also showed the fragment ion at *m*/*z* 811, grouped in a cluster containing three fragment ions, which may correspond to oligomers having the same degree of polymerization but having a different a content of HB and HV units. The fragmentation results allowed us to confirm that in the presence of O-PEW carbon source the PHA formed was composed of 3-hydroxybutyrate, 3-hydroxyvareate as well as 3-hydroxyhexanoate co-monomer units which are randomly distributed along the oligomer chain [34,38,40]. Moreover, with respect to the hitherto reported studies, this research demonstrated the possibility of PHA structure evaluation even when the content of HV and HH is lower than 5%, *i.e.*, on the level of accuracy of ^1^H-NMR measurements [34].

The structure of the biopolyesters obtained influences their thermal properties, especially the glass transition temperature (*Tg*) and melting transition (*T_m_*). Both of these values were found to be lower in the case of the biopolyester obtained when O-PEW was used as a carbon source (Table 2). In the case of *Tg* the difference is equal to 4.1 °C, whereas in case of *Tm* the difference is equal to 5 °C. Differences are also visible in crystallinity degree, and PHA derived from fermentation with the use O-PEW is less crystalline. Thus, even three per cent of repeating unit with longer aliphatic chains in *β*-position may influence significantly on *Tg*, *Tm* and the degree of crystallinity of PHA obtained.

## 4. Materials and Methods

### 4.1. Carbon Source

Oxidized wax from PE standard samples was used as a carbon source for the PHA production via small scale shake flasks experiments. Oxidized PE waxes were obtained as products from oxidative degradation which was carried out in a two-phase system: gas-liquid phase, after melting at 145 °C and using oxygen. The raw material used for this process was a polyethylene standard from Sigma-Aldrich Co (Gillingham, UK). with an average *M_w_* ~4000 and an average *M_n_* ~1700 (by GPC), melting point 92 °C, soft point 106 °C, and density 0.92 g/mL at 25 °C.

To increase the oxidation rate and in order to obtain products with high values of acidity number (AN), ozone (100–300 mg/L) was introduced into the oxygen stream. Processes were carried out from 18 to 20 h.

The acid number (AN) which is one of the basic properties of oxidized waxes was determined according to standard methods [42]. Each analysis was performed a minimum of two times. The obtained results of analysis for the same wax were very similar and the differences for AN were less than 1.0 mg KOH/g. The standard allow for 3% measurement accuracy.

### 4.2. Microorganism

The microorganism used for the PHA production with oxidized polyethylene wax was *R. eutropha* H16 (NCIMB 10442, ATCC 17699). The organism was obtained from the National Collection of Industrial and Marine Bacteria, Aberdeen, UK. The stock culture was freeze-dried and kept at −20 °C. Before use, cultures were resuscitated and grown overnight at 30 °C in tryptone soya broth (TSB). The organism was then sub-cultured on tryptone soya agar (TSA) and incubated at 30 °C for 24 h.

### 4.3. Growth Media and Chemicals

Tryptone soya broth (TSB) and tryptone soya agar (TSA) were purchased from Lab M Ltd., Lancashire, UK. Both media were prepared following the instructions of the manufacturer under aseptic conditions. Basal salts medium (BSM) contains distilled water, 1 g/L K_2_HPO_4_, 1 g/L KH_2_PO_4_, 1 g/L KNO_3_, 1 g/L (NH_4_)_2_SO_4_, 0.1 g/L MgSO_4_·7H_2_O, 0.1 g/L NaCl, and 10 mL/L trace elements solution. Trace element solution has: 2 mg/L CaCl_2_, 2 mg/L CuSO_4_·5H_2_O, 2 mg/L MnSO_4_·5H_2_O, 2 mg/L ZnSO_4_·5H_2_O, 2 mg/L FeSO_4_, and 2 mg/L (NH_4_)_6_Mo_7_O_24_·4H_2_O. BSM salts were purchased from BDH Chemicals Ltd., Poole, UK. Ringer’s solution was used for microbial growth analysis and it was also purchased from Lab M. A 1/4 strength tablet was used in distilled water. All media were sterilized by autoclaving at 121 °C for 15 min. Chloroform and *n*-hexane (High-performance liquid chromatography (HPLC) grade) used for PHA extraction and precipitation were obtained from Sigma Aldrich, Gillingham, UK.

### 4.4. Shake Flask Experiments

For the small scale shake flasks studies, starter cultures were prepared by inoculating 20 mL TSB with single colonies of *R. eutropha* H16 in 50 mL conical flasks. The culture was then incubated aerobically for 24 h at 30 °C and 150 rpm in a rotary incubator (New Brunswick Scientific Co. Series 25 Incubator Shaker, Enfield, CT, USA) After 24 h of incubations, cultures were checked for purity by Gram staining and microscopic observations at a magnification of ×1000.

Shake flasks studies were performed in triplicate using 500 mL Erlenmeyer flasks. 1.25 g of the wax was put into a 50 mL beaker covered with foil paper and melted at 70 °C. 20 mL sterile TSB or BSM was then added to the melted wax which caused the wax to solidify again as expected. Thus, the temperature was gradually increased until all the wax had melted again. After which the wax suspension was sonicated for 7 min at 0.5 active and passive intervals with a power of 60% using a Bandelin Sonopuls HD2070 sonicator, Berlin, Germany. The TSB/wax emulsion or BSM/wax emulsion was tested for sterility by spread plating 100 mL of the emulsion on TSA plates overnight at 37 °C and 30 °C. No microbial growth was observed in either plate after 24 h of incubation, thus indicating that the emulsion was sterile.

The emulsion was then added to 210 mL sterile TSB or BSM in a 500 mL Erlenmeyer flask and this was immediately followed by the addition 20 mL starter culture, giving a total volume of 250 mL with a wax concentration of 4 g/L and 8% (*v*/*v*) of the starter culture. For the control experiment, 230 mL TSB and BSM was inoculated with 20 mL starter culture with no wax. This was done to see the effect of the wax on both the growth of cells and the production of PHA by *R. eutropha* H16 cells. All flasks were incubated in a rotary incubator for 48 h at 30 °C and 150 rpm.

5 mL samples were aseptically collected from the growing bacterial cultures at 0, 3, 6, 24, and 48 h of incubation for viable cell counts. Cell counts were carried out using the method described by Miles and Misra [43] involving a serial dilution of the samples to 10^−8^ and aseptically inoculating 20 µL of each dilution unto TSA plates in triplicate. Plates were incubated overnight at 30 °C, followed by colony counting. The results obtained were expressed in log_10_ CFU/mL.

### 4.5. PHA Extraction

PHA extraction was carried out as described previously [31]. Shake flask experiments were left to run for 48 h after which microbial experiments were stopped and cultures were centrifuged in a Hermle Labortechnik (Wehingen, Germany) Z300K centrifuge for 15 min at 6000 rpm. Supernatants were discarded and the biomass was obtained and frozen overnight at −20 °C. This was followed by lyophilization using an Edwards Modulyo freeze-drier (Crawley, UK) for 72 h at a temperature of −40 °C and at a pressure of 5 mBar. The weights of the dry biomass were obtained and recorded as cell dry weight (CDW). Lyophilized biomass was then transferred into extraction thimbles and PHA was extracted by Soxhlet extraction with HPLC grade chloroform (Sigma Aldrich) running for 5 h. The hot solution of polymer/chloroform was concentrated by evaporation, followed by PHA precipitation in *n*-hexane (Sigma Aldrich). Precipitated polymer was separated from solution by filtration (Watman No. 1 paper, Sigma Aldrich) and rinsed further in chloroform to remove the excess wax materials before air-drying. The weight of the polymer (*W_PHA_*) was recorded and the percentage of PHA (*wt*/*wt*) synthesized by *R. eutropha* H16 was calculated using the equation:

$$PHA\%=\frac{W_{PHA}}{CDW}\times 100$$

### 4.6. Polymer Identification

#### 4.6.1. GPC analysis

The number-average molar mass (*M_n_*) and the molar mass distribution index (*M_w_*/*M_n_*) of the plain PHA samples were determined by GPC experiments conducted in a CHCl_3_ solution at 35 °C with a flow rate of 1 mL/min using a Spectra-Physic 8800 solvent delivery system (Spectra-Physics Inc., San Jose, CA, USA). This system had a set of two PLgel 5 µm MIXED-C ultra-high efficiency columns and a Shodex SE 61 refractive index detector. A sample solution volume of 10 µL (concentration of 1% *w*/*v*) was injected. Polystyrene (PS) standards with a narrow molar mass distribution were used to generate calibration curves. The PS with nominal *Mp* in the range of 3000–7,000,000 g/mol were used.

#### 4.6.2. NMR Analysis

^1^H-NMR spectra were recorded with a Bruker Avance II (Bruker, Rheinstetten, Germany) operating at 600 MHz, with 64 scans, 2.65 s acquisition time and 11 µs pulse width. ^13^C-NMR spectra were recorded with a Bruker Avance II operating at 150.9 MHz, with 20,480 scans, 0.9088 s acquisition times, and 9.40 µs pulse width. ^1^H-NMR and ^13^C-NMR spectra were run in CDCl_3_ at room temperature with tetramethylsilane (TMS) as an internal standard.

#### 4.6.3. ESI-MS/MS Analysis

Electrospray mass spectrometry analysis was performed using a Finnigan LCQ ion trap mass spectrometer (Thermo Finnigan LCQ Fleet, Thermo Fisher Scientific Inc., San Jose, CA, USA). The oligomer samples, prepared as described in [33], were dissolved in a chloroform/methanol system (1:1 *v*/*v*), and the solutions were introduced into the ESI source by continuous infusion using the instrument syringe pump at a rate of 5 μL/min. The LCQ ESI source was operated at 4.5 kV, and the capillary heater was set to 200 °C. Nitrogen was used as the nebulizing gas. For ESI-MS/MS experiments, the ions of interest were isolated monoisotopically in the ion trap and were collisionally activated. The helium damping gas that was present in the mass analyzer acted as a collision gas. The analysis was performed in the positive-ion mode.

#### 4.6.4. DSC Analysis

DSC analyses were taken with a TA DSC 2010 apparatus (TA Instruments, New Castle, DE, USA) in the temperature range of −50 to +200 °C and the samples were run in triplicate. The glass transition temperatures (*Tg*) were determined at a heating rate of 20 °C/min. In this study, *Tg* was taken as the midpoint of the step-transition. The instrument was calibrated with high purity indium and gallium.

Percentage crystallinity was calculated by the equation:

$$C\left(\%\right)=\frac{\Delta H_{m}^{m}}{\Delta H_{m}^{100\%}}$$

where 𝐶 is the percentage crystallinity; $\Delta H_{m}^{m}$ is the measured melting enthalpy (J/g); and the $\Delta H_{m}^{100\%}$ is the melting enthalpy for fully crystalline PHB (146 J/g) [44].

#### 4.6.5. TGA Analysis

Thermogravimetric analysis was performed using a Mettler-Toledo TGA/DSC1 unit. Analyses were performed under an inert atmosphere (nitrogen) at a gas flow rate of 60 mL/min. The rate of temperature increase during the analysis was 10 °C/min. The measurements were performed in the ceramic pan capacity of 70 uL to weighed amounts of approximately 10 mg. The samples were run in triplicate.

## 5. Conclusions

It may be concluded, that oxidized polyethylene wax can be a promising carbon source for PHA production. The structural and thermal characterization of biopolyesters obtained revealed that using TSB only or TSB supplemented with O-PEW as a carbon source led to the production of different biopolyesters. We have, therefore, demonstrated uniquely that the addition of oxidized polyethylene waxes to the fermentation medium can have an influence on the structure of resulting biopolyesters and, hence, their thermal properties. Growth on O-PEW in TSB not only enhances PHA yield but also influences compositional changes in the PHA polymer structure.

The NMR analysis indicated that in the presence of TSB/O-PEW the chains of the resulting PHA biopolyester containing not only 3-hydroxybutyrate repeating units. However, it was not possible to determine the precise structure of this biopolyester with the aid of this technique. Nonetheless, based on the integration of particular signals in the ^1^H-NMR spectrum of the polyester obtained from TSB/O-PEW the content of 97 mol % of 3-hydroxybutyrate units and approximately 3 mol % of another aliphatic repeating units were estimated.

The ESI-MS as well as tandem ESI-MS/MS techniques confirmed that when O-PEW is used as a carbon source, besides the 3-hydroxybutyrate repeating units the units containing longer aliphatic moieties in the *β*-position are formed. The ESI-MS/MS fragmentation experiments confirmed the presence of 3-hydroxyvalerate repeating units as well as 3-hydroxyhexanoate repeating units in the biopolyester obtained in the presence of O-PEW as a carbon source. Thus, this analytical technique enables the PHA characterization at the molecular level even when the content of 3-hydroxybutyrate is high and the appearance of other PHAs repeating units is very low.

## Figures and Tables

**Table 1:** PHA production by *Ralstonia eutropha* using 5 g/L PE wax, during 48 h of incubation in TSB, TSB with O-PEW and BSM with O-PEW at 30 °C. Presented values are the means of triplicates.

Media	Average CDW (g/L)	Average PHA (g/L)	PHA (% *w*/*w*)
TSB only	2.24 ± 0.05	0.39 ± 0.05	17.00%
TSB with O-PEW	3.66 ± 0.20	1.24 ± 0.10	33.80%
BSM with O-PEW	1.85 ± 0.11	ND	ND

ND—no detected.

**Table 2:** Selected thermal properties of the obtained polyesters.

Sample	*T_g_* (°C)	*T_m_* (°C)	∆*H_m_* (J/g)	*C* (%)	*T*_max_ (°C)
PHB from TSB	6.2	171	68.9	47	296
PHA from TSB/O-PEW	1.9	166	61.8	42	290

## Abbreviations

The following abbreviations are used in this manuscript:

PE	Polyethylene
PHA	Polyhydroxyalkanoates
PHB	Poly 3-hydroxybutyrate
O-PEW	Oxygenated Polyethylene Wax
TSB	Tryptic Soy Broth
ESI	Electrospray ionisation
HB	3-hydroxybutyrate
HV	3-hydroxyvalerate
HH	3-hydroxyhexanoate
TMS	Tetramethylsilane
